# Bayesian Modeling and Estimation of Spatial Risk for Hospitalization and Mortality from Ischemic Heart Disease in Paraná, Brazil

**DOI:** 10.5334/gh.1347

**Published:** 2024-08-05

**Authors:** Amanda de Carvalho Dutra, Lincoln Luis Silva, Amanda Gubert Alves dos Santos, Rogério do Lago Franco, Giane Aparecida Chaves Forato, Marcela Bergamini, Isadora Martins Borba, Edvaldo Vieira de Campos, Catherine Ann Staton, Diogo Pinetti Marquezoni, Oscar Kenji Nihei, João Ricardo Nickenig Vissoci, Luciano de Andrade

**Affiliations:** 1 Post-Graduation Program in Health Sciences, State University of Maringa, Parana, Brazil; 2 Department of Emergency Medicine, Duke University School of Medicine, Durham, North Carolina, United States of America; 3 Centro Universitário Integrado, Campo Mourao, Parana, Brazil; 4 Department of Medicine, State University of Maringa, Maringa, Parana, Brazil; 5 Regional University Hospital of Maringa, State University of Maringa, Parana, Brazil; 6 Duke Global Health Institute, Durham, North Carolina, United States of America; 7 Hospital das Clinicas of the Medical School of Botucatu, UNESP, Sao Paulo, Brazil; 8 Education, Letters and Health Center, State University of the West of Paraná, Foz do Iguaçu, Paraná, Brazil

**Keywords:** spatiotemporal analysis, ischemic heart disease, Bayesian analysis, epidemiology

## Abstract

**Objective:**

Despite significant advancements in understanding risk factors and treatment strategies, ischemic heart disease (IHD) remains the leading cause of mortality worldwide, particularly within specific regions in Brazil, where the disease is a burden. Therefore, the aim of this study was to estimate the risk of hospitalization and mortality from IHD in the state of Paraná (Brazil), using spatial analysis to identify areas with higher risk based on socioeconomic, demographic and health variables.

**Methods:**

This is an ecological study based on secondary and retrospective IHD hospitalization and mortality data obtained from the Brazilian Hospitalization and Mortality Information Systems during the 2010–2021 period. Data were analyzed for 399 municipalities and 22 health regions in the state of Paraná. To assess the spatial patterns of the disease and identify relative risk (RR) areas, we constructed a risk model by Bayesian inference using the R-INLA and SpatialEpi packages in R software.

**Results:**

A total of 333,229 hospitalizations and 73,221 deaths occurred in the analyzed period, and elevated RR of hospitalization (RR = 27.412, CI 21.801; 34.466) and mortality (RR = 15.673, CI 2.148; 114.319) from IHD occurred in small-sized municipalities. In addition, medium-sized municipalities also presented elevated RR of hospitalization (RR = 6.533, CI 1.748; 2.006) and mortality (RR = 6.092, CI 1.451; 2.163) from IHD. Hospitalization and mortality rates were higher in white men aged 40–59 years. A negative association was found between Municipal Performance Index (IPDM) and IHD hospitalization and mortality.

**Conclusion:**

Areas with increased risk of hospitalization and mortality from IHD were found in small and medium-sized municipalities in the state of Paraná, Brazil. These results suggest a deficit in health care attention for IHD cases in these areas, potentially due to a low distribution of health care resources.

## Introduction

Ischemic heart disease (IHD) is a cardiovascular disease (CVD) that imposes a significant global public health issue, responsible for over 9 million deaths annually (1,2,3). IHD deaths are influenced by various non-clinical factors (4). For example, regions with lower sociodemographic indices present higher burden of IHD (4,5,6). In addition, age-standardized IHD mortality rates have declined in many high-income countries, and IHD mortality rates in the working age population are higher in low-and middle-income countries than in high-income countries (7).

In Brazil, data from 2017 showed that IHD remains the major cause of morbidity and mortality, accounting for more than 170 thousand deaths annually (8). Additionally, IHD contributed to 32.1% of fatalities related to cardiovascular diseases in 2019 (9). However, the distribution of this disease varies across Brazilian states, with a greater prevalence observed in regions characterized by lower socioeconomic development. However, even within more developed states, the prevalence of IHD is worrying, and risk factors need to be investigated (10). Hence, there is a need to investigate what causes the problem locally.

Spatiotemporal modeling has emerged as a valuable approach to understanding the geographical distribution and temporal patterns of IHD and its risk factors (11). Studies have shown that spatial analysis can provide insight on IHD cases and identify high-risk areas, aiding in the development of targeted interventions and resource allocation strategies (12). Additionally, the integration of Bayesian modeling techniques has been instrumental in capturing the spatial and temporal variations of IHD, allowing for the identification of significant risk factors and the assessment of their impact on disease occurrence (13). Such approaches have been successfully applied in diverse settings, including the analysis of regional disparities in IHD prevalence and the evaluation of the effectiveness of intervention programs (11,14,15). These studies highlight the utility of spatial analysis in detecting spatial patterns, assisting policy decisions, and improving the understanding of IHD epidemiology.

Identifying the regions most impacted by IHD and the socioeconomic, demographic and health variables that increase the risk of the disease is a crucial step towards developing effective prevention and control strategies. However, despite the relevance of this information, studies that locally quantify the relative risk of each variable are still scarce. Given this gap, this study proposes to: (i) estimate the risk of hospitalization and mortality due to IHD in the state of Paraná, Brazil, and (ii) identify areas with higher risk of hospitalization and mortality based on socioeconomic, demographic and health variables.

## Methods

### Study design and location

This is an ecological study based on publicly available secondary retrospective data. The research was conducted in the state of Paraná, in southern Brazil, using data of individuals aged 40 years and above, from 2010 to 2021. Methodological quality was ensured by following the recommendations of the Strengthening the Reporting of Observational Studies in Epidemiology (STROBE) statement (16).

**Figure 1:**
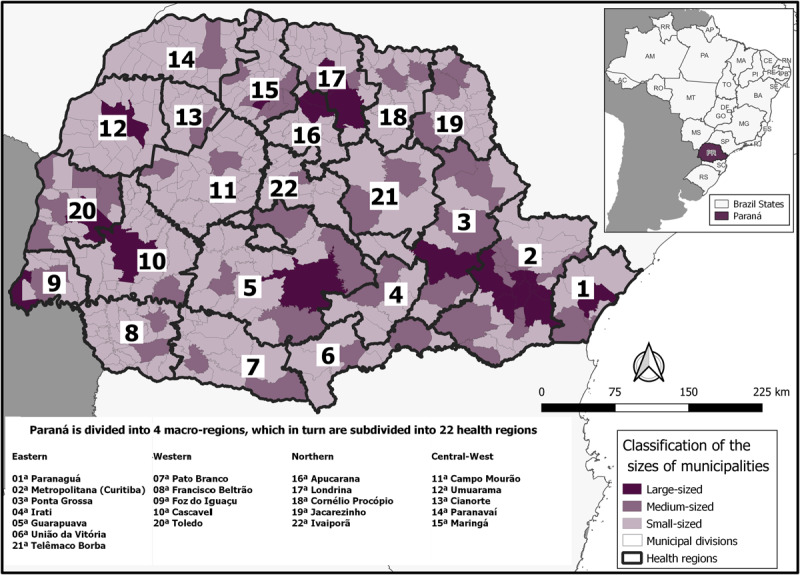
Brazil and its States with the Paraná highlighted in purple, alongside the 22 Health Regions of Paraná and location of their municipalities according to population size classification (2021). The shapefile for the basemap was obtained from the Brazilian Institute of Geography and Statistics (17).

Paraná is the fifth most populous state in the country, with 11.4 million people distributed across 399 municipalities, according to data from the Brazilian Institute of Geography and Statistics (IBGE) (17). The 2022 demographic census showed that 81% of Paraná’s municipalities are small-sized (<25 thousand people), 14% are medium-sized (25–100 thousand people), and 5% are large-sized (>100 thousand people) (17). Paraná has the seventh highest Human Development Index (HDI) in Brazil, at a value of 0.769 (17). In this study, data were collected from 399 municipalities, which were then aggregated according to the state’s 22 Health Regions (Figure 1).

Health Regions are defined based on geographical proximity and population coverage, aiming to ensure equitable access to health care services (18,19). These regions play a crucial role in the coordination and integration of health care actions, as well as in the allocation of resources and the implementation of health policies (20). Additionally, health regions serve as a basis for epidemiological surveillance, enabling the monitoring of disease patterns and the identification of health inequalities across different geographical areas (21,22). The use of Health Regions as units of analysis in research allows for a comprehensive understanding of the spatial distribution of health outcomes and the evaluation of health care interventions within specific geographic contexts (21,22).

In this study, we utilized data from individuals aged 40 years and above, as this demographic is known to face a higher risk of cardiovascular disease (23,24).

### Data sources

Demographic data and basemaps were collected from the Brazilian Institute of Geography and Statistics (IBGE) (17). Hospitalization and mortality data were obtained from the Hospitalization Information System (SIH) and Mortality Information System (SIM), respectively, and both systems are publicly and freely accessible through the Information Technology Department of the Unified Health System (DATASUS) (25).

Basemaps and population numbers for each municipality were sourced from IBGE and are also open to the public (26). According to the 2022 Continuous National Household Sample Survey conducted by the IBGE in Paraná, out of the individuals who declared their demographics, 48.9% were men and 51.1% were women. Furthermore, 35.6% fell within the age range of 40 to 80 years old. Specifically, 24.4% were aged between 40–59 years, 6.4% between 60–69 years, 3.4% between 70–79 years, and 1.4% were above 80 years old (27). Racial composition comprised 70.3% White, 3.2% Black, 1.2% Yellow, 25.1% Brown, and 0.2% Indigenous, where ‘Brown’ encompasses diverse backgrounds (26,28). For this study, we simplified the race categories into two groups: White and Non-White, the latter including the combined percentages of Black, Yellow, Brown, and Indigenous populations.

Municipal Performance Index (IPDM) for all municipalities in Paraná were collected from the Institute of Economic and Social Development of Paraná (IPARDES) (29). The IPDM, developed by the IPARDES, integrates income, health, and education data seamlessly, utilizing official statistics from publicly available administrative records updated annually. This index, ranging from 0 to 1, assesses municipality-level performance, with higher values indicating better performance. Municipalities are categorized into four groups based on their index values: low (0 to < 0.4), low-medium (0.4 to < 0.6), medium (0.6 to < 0.8), and high (0.8 to 1) (29). Table 1 presents the source for all datasets accessed and utilized in this study.

**Table 1:** Data sources for analysis.


SOURCE	VARIABLES	REFERENCE

DATASUS – Mortality Information System (SIM)	Mortality sex (Men, Women), race (White, Non-White) Age groups (40–59, 60–69, 70–79, ≥80)	(25)

DATASUS – Hospitalizations Information System (SIH)	Hospitalizations sex (Men, Women), race (White, Non-White) Age groups (40–59, 60–69, 70–79, ≥80)	(25)

IBGE – Brazilian Institute of Geography and Statistics	Population by sex (Men, Women), race (White, Non-White) Age groups (40–59, 60–69, 70–79, ≥80) Municipality Size of the municipality’s population Basemap for all municipalities and regional health in Paraná	(26)

IPARDES – Institute of Economic and Social Development of Paraná	Municipal Performance Index (IPDM)	(29)


### Outcome variables

**Figure 2:**
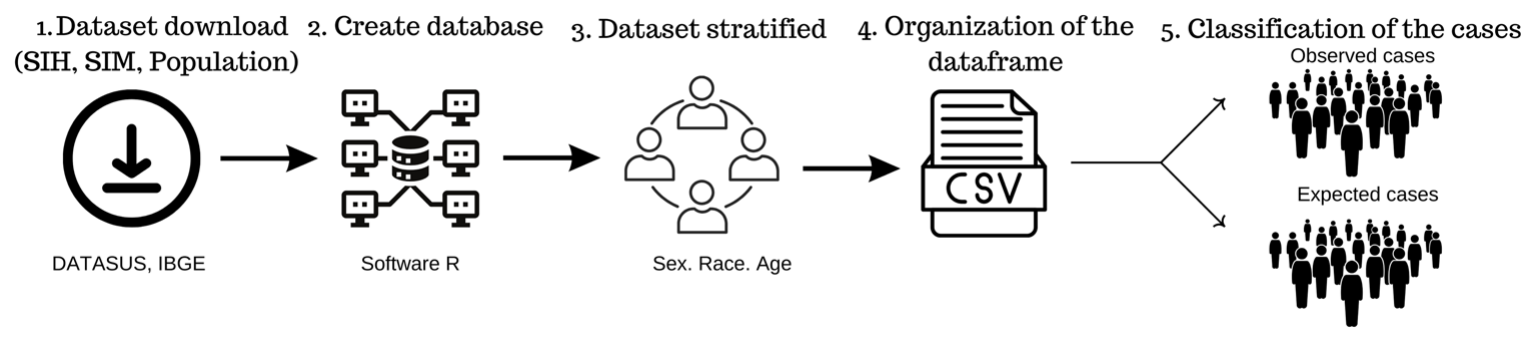
Structure of the ischemic heart disease dataset stratified by sex, race, and age group for the development of the statistical model.

In this study, we utilized two outcome variables: Standard Incidence Ratio (SIR) and Standard Mortality Ratio (SMR). Both were derived from the total number of hospitalizations and mortality, respectively, filtered for cases related to IHD according to the International Classification of Diseases (ICD), ranging from ICD-X I20 to I25. Specifically, I20 pertains to all notifications of Angina Pectoris, I21 to Acute Myocardial Infarction, I22 to Recurrent Myocardial Infarction, I23 to some current subsequent complications of Acute Myocardial Infarction, I24 to Other Acute Ischemic Heart Diseases, and I25 to Chronic Ischemic Heart Disease. These data were grouped by sex (female and male), race (White and non-White), and age group (40–59, 60–69, 70–79, and ≥80 years old), as shown in the flowchart of Figure 2. After filling out the form, a data frame was created with the observed and expected cases of hospitalization and mortality for each municipality in order to commence the statistical analysis.

### Statistical analysis

#### Population analysis

We focused our study on individuals aged 40 and above because they have been identified in several studies as having a heightened risk of IHD (23,24). Hence, we conducted an analysis comparing hospitalization and deaths numbers between two age groups: those over 40 and those under 40. We observed a greater number of hospitalizations and deaths in the group aged 40 and above. Using independent samples t-tests, we confirmed statistically significant differences (p < 0.05) for both hospitalizations and deaths, indicating higher numbers in the group aged 40 and above compared to those under 40. Therefore, we concluded that individuals aged 40 and above experience a statistically significant increase in hospitalizations and deaths due to IHD compared to younger individuals.

#### Spatial modeling using Inference for Latent Gaussian Models (INLA)

Bayesian inference is a useful method for data modeling as it allows us to estimate the posterior distributions of parameters (30). This process involves updating the initial information, represented by prior distributions, based on the information from the observed data using Bayes’ theorem. In this study, we used Inference for Latent Gaussian Models (INLA) (30), which is a Bayesian approach, to model the incidence and mortality rates based on a covariate. INLA enables us to perform inference in spatial models and adjust the rates according to the spatial structure of the data. This requires calculations for the Standard Incidence Ratio (SIR), Standard Mortality Ratio (SMR) and Relative Risk (RR). This way, we will obtain risk values for each municipality (31). We used the Bernardinelli model formula for this purpose (32). This model assumes that the number of observed cases in a specific municipality each year follows a Poisson distribution. For further details on the methods used in this study, please refer to the supplementary material, which includes the formulas and additional information.

#### SIR, SMR, and RR Analysis

Data obtained from SIH and SIM were used to calculate the expected number of IHD hospitalizations and deaths per year over a total of 12 years. The Standardized Incidence Ratio (SIR) is used to evaluate disease incidence by comparing the number of observed cases with the number of expected cases, considering a reference population. An SIR greater than 1 indicates an observed incidence rate higher than the expected rate in the study population. The Standardized Mortality Ratio (SMR) is used to assess mortality in a population compared to a reference population by calculating the ratio of observed deaths to expected deaths. An SMR greater than 1 indicates an observed mortality rate higher than the expected rate in the study population (33).

We used a method called the ‘expected’ function from the SpatialEpi package of the R software to calculate the expected values. This function took into account various factors, such as population size and the number of hospitalizations and deaths, and stratified the data into 16 strata based on race, sex and age groups. Then, SIR and SMR values were generated for each municipality and health region of Paraná (34). SIR and SMR results provide insight into the risk for IHD-related events in Paraná; however, such information may be misleading or insufficiently dependable in areas with small populations. A solution to this limitation is given by model-based approaches that include covariates and information from neighboring municipalities to improve local estimates, resulting in the smoothing of values for areas with small sample sizes (34).

Another parameter used in the structure of our model is the application of the Besag, York, and Mollié model (BYM) to estimate the RR and identify high- or low-risk areas (35). RR is a measure that compares the risk of an event between distinct populations; it assesses the relationship between the probability of event occurrence in an observed population compared to an expected population. An RR greater than 1 indicates an increased risk in the exposed population. By combining these measures, we can obtain valuable information about the health of the population and identify areas or groups with elevated risks, thereby guiding disease prevention and control strategies. RR estimates were obtained by calculating posterior means, and their uncertainty was obtained using 95% credibility intervals (95% CI) (35).

#### Geographic variables and municipal development index in the INLA model

We included IPDM and size of the municipality’s population as covariates to examine their relationship with SIR and SMR. Additionally, we conducted an exploratory analysis to detect outliers by examining the association between the response variable and covariates, as well as evaluating collinearity among the covariates. To accomplish this, we calculated the variance inflation factors (VIF) using Poisson regression and removed variables in case of exceeding 5 as indicative of collinearity (36,37).

We considered a variable statistically significant if its estimated coefficient mean was positive and both the lower and upper limits of the CI were positive, or vice versa (30). The relative risk was evaluated by applying the exponential function to the mean. The results of SIR, SMR and RR were plotted to understand the spatial patterns of the disease and identify risk areas. The procedures used in INLA were those described in the R-INLA package, R Statistical Software (v4.1.2; R Core Team 2021). Choropleth maps were constructed using the QGIS software version 3.16 (38). Further details on the modeling methodology can be found in the Supplementary Material (https://doi.org/10.6084/m9.figshare.25007108.v1).

### Ethical aspects

This study was approved by the Research Ethics Committee (CAEE) at the State University of Maringá (process No. 34478620.1.0000.0104).

### Data availability

The database and script utilized in this study can be found in the online repository: https://doi.org/10.6084/m9.figshare.25007108.v1.

## Results

Between 2010 and 2021, there were 333,229 hospitalizations and 73,221 deaths attributed to IHD among individuals aged over 40 years in the state of Paraná. Demographic stratification revealed a higher number of hospitalizations among men (178,419–53%), White individuals (282,900–85%), and individuals aged 40–59 years (127,124–38%). Similarly, the number of deaths followed the same pattern, with a higher number observed among men (41,269–56%) and White individuals (59,319–81%). This trend was also observed among non-White women aged 60–69 years (2,055 deaths–34% of deaths in this age group) and 70–79 years (1,821 deaths–30% of deaths in this age group). An exception to this pattern was found in individuals aged 80 years and older (7,672–22%), where mortality rates were higher among white women compared to White men.

**Figure 3:**
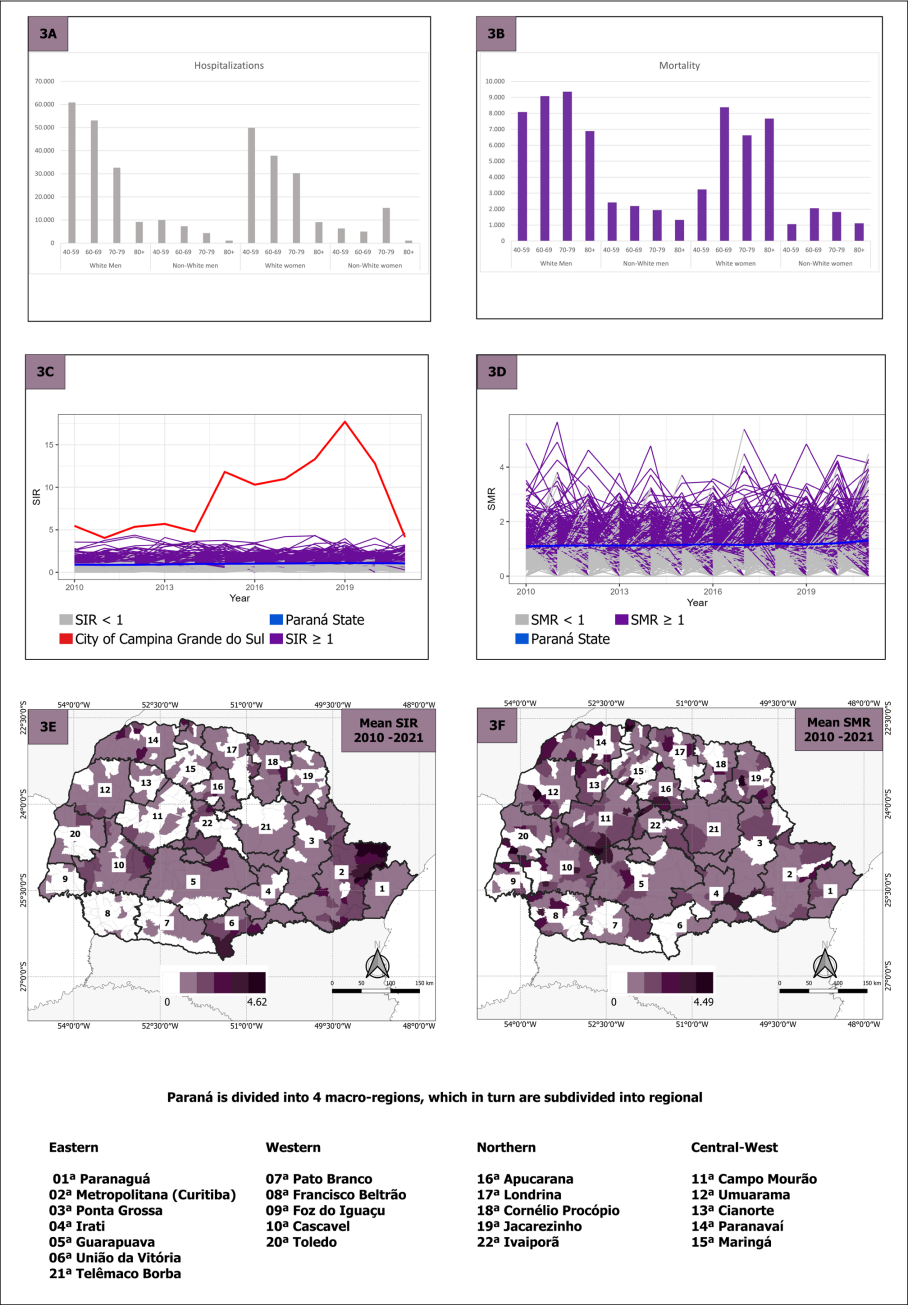
**3A** Absolute values of hospitalization from ischemic heart disease. **3B** Absolute values of deaths from ischemic heart disease. Panels **3C** and **3D** present the temporal distribution of the standardized incidence ratio (SIR) and standardized mortality ratio (SMR) of ischemic heart disease (IHD) in municipalities of Paraná from 2010 to 2021. Panels **3E** and **3F** show the geographic distribution of the mean SIR and SMR for IHD, in the municipalities of Paraná from 2010 to 2021. The shapefile for the basemap was obtained from the Brazilian Institute of Geography and Statistics (17).

Figure 3 presents the absolute frequency of hospitalization and mortality due to IHD. A notable decrease in hospitalizations is observed in Figure 3A with advancing age among both White women and men of White and non-White races. However, a peak in hospitalizations is evident within the age range of 70 to 79 years for non-White women. Regarding mortality, Figure 3B shows a shift in patterns, with two peaks in mortality for white women (between 60 to 69 years and over 80 years) and one peak for white men (70 to 79 years). Additionally, Figure 3C and 3D depict the time series of Standardized Incidence Ratios (SIR) and Standardized Mortality Ratios (SMR) for the 399 municipalities of Paraná, highlighting significant variations in certain regions over time. In Panel 3C, we observe that Campina Grande do Sul exhibited higher variation between 2014 and 2019, followed by a sharp decline from 2020 onwards, approaching the state’s average. In Panel 3D, we observe variations of SMR over time. Figure 3E and 3F present choropleth maps depicting SIR and SMR, where higher SIR values were observed in the eastern region of the state, specifically in the Metropolitan Health Region of Curitiba and Paranaguá. Regarding SMR, the rates are diffuse, meaning they vary throughout the state, but with a predominance in the central region.

In Table 2, both SIR and SMR indicate a positive association between the smaller size of the municipality and higher mean values, suggesting increased risk of cases and death of IHD. Positive association was also observed between these variables and the medium size-municipalities. Additionally, IPDM showed a negative value with IHD hospitalizations (SIR) and mortality (SMR), indicating that regions with a lower index will be more likely to have a higher number of IHD hospitalization and mortality. To assess the RR, we can use the exponential value of the mean. Specifically, small-size municipalities have a RR of SIR equal to 27.412 and SMR equal to 15.673, while medium-size municipalities have a RR of SIR equal to 6.533 and SMR equal to 6.092, compared to large-size municipalities. IPDM presents an RR close to 0, indicating a weak correlation between IPDM and SIR/SMR. Additionally, considering the WAIC and Marginal log-Likelihood, both models demonstrated good performance, implying that they are suitable for explaining the results of both SIR and SMR. These parameters were not used to compare SIR and SMR. However, it is important to be expressed once further studies in the region can be assessed to compare the progress of the covariates in the following years.

**Table 2:** Summaries of the probability distributions of the covariate coefficients for Standardized Incidence Rate (SIR) and Standardized Mortality Rate (SMR).


COVARIATES	SIR			SMR		
	
	MEAN	95% CI	RR	MEAN	95% CI	RR

Intercept	–6.875	–7.334; –6.415	–	–5.716	–7.002; –4.430	–

IPDM	–8.274	–8.859; –7.689	0.000	–9.824	–11.437; –8.210	0.000

Small-size municipalities	3.311	3.082; 3.540	27.412	2.752	0.765; 4.739	15.673

Medium-size municipalities	1.877	1.748; 2.006	6.533	1.807	1.451; 2.163	6.092

Large-size municipalities	–	–	–	–	–	–

Marginal log-Likelihood	–880.98			–182.91		

WAIC	1,198.2			299.3		


95% CI: 95% credibility interval. RR: Relative risk.

**Figure 4:**
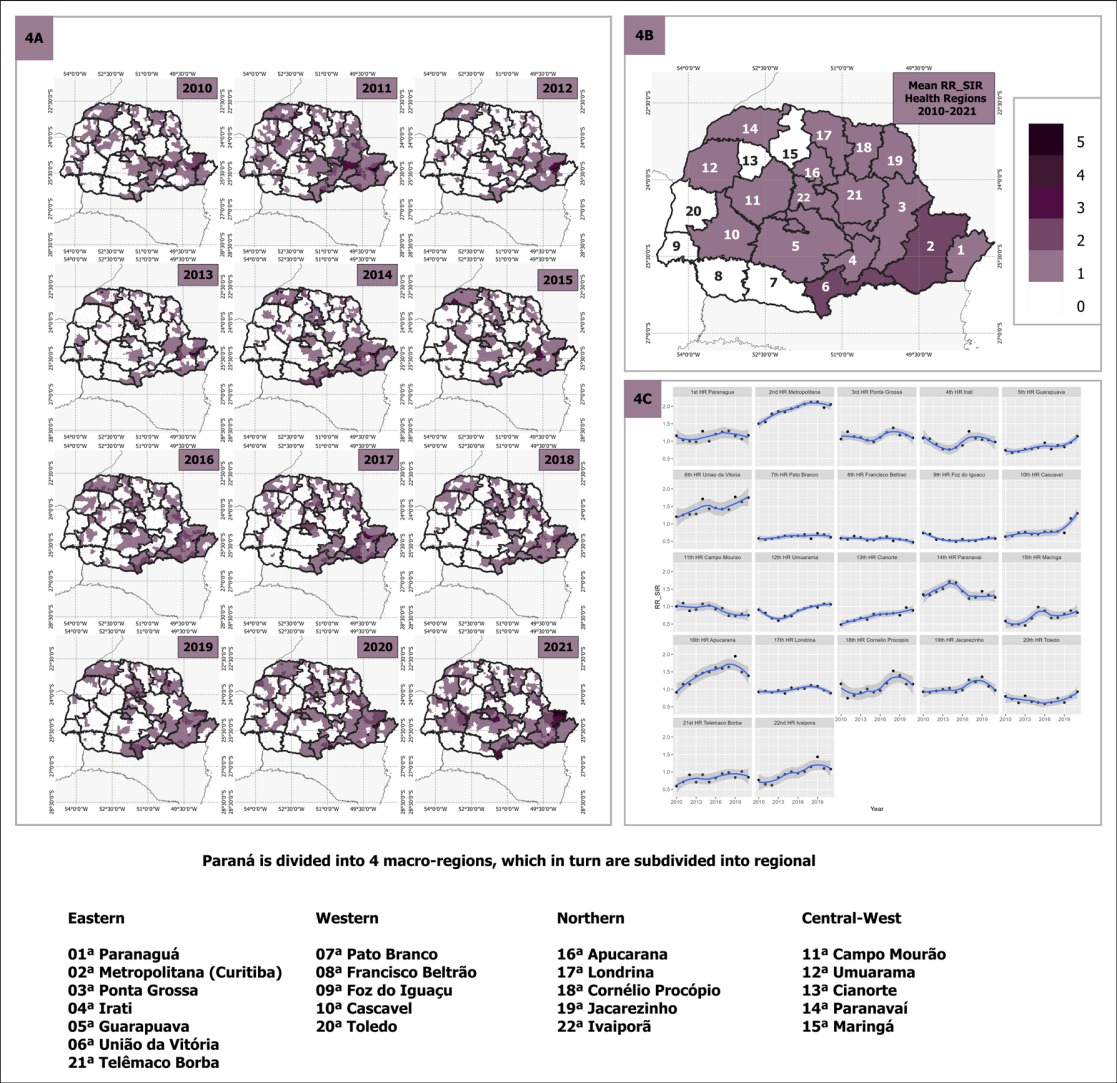
**4A** Relative risk estimates of standardized incidence ratio (SIR) per municipality and health region and scatter plot of SIR RR values per health region from 2011 to 2021 in Paraná. **4B** Average SIR RR in municipalities by health region from 2010 to 2021. **4C** Graphs with temporal presentation of average SIR RR in municipalities by health region, from 2010 to 2021. The shapefile for the basemap was obtained from the Brazilian Institute of Geography and Statistics (17).

Figure 4A presents the spatial distribution of RR for hospitalization over time from 2010 to 2021. This figure highlights significant variations in RR across different regions throughout the analyzed period. Municipalities with an RR greater than 1 had higher hospitalization risk than expected, while regions with values lower than 1 had lower risk than expected. The shading on the map indicates the intensity of the RR, with darker regions representing a RR greater than 1. In Figure 4A, a diverse regional pattern of hospitalization rates is evident, indicating significant variations across different areas. This suggests that some regions are more vulnerable to hospitalization than others. Moving to Figure 4B, we see the aggregated mean of RR for each health region, which provides a broader overview of the regional risk distribution. It is evident from this data that regions 2 and 6 are the most affected, showing higher hospitalization risk compared to other areas. Figure 4C presents the trend of RR over the years for each health region. Several regions (1, 2, 3, 5, 6, 10, 12, 14, 16, 18 and 22) exhibited RR values greater than 1 in recent years. However, regions 5, 6, 10, 12, 13, 20 demonstrated a positive trend of increasing hospitalizations. This indicates that special attention is needed in these specific regions to address the rising hospitalization rates.

**Figure 5:**
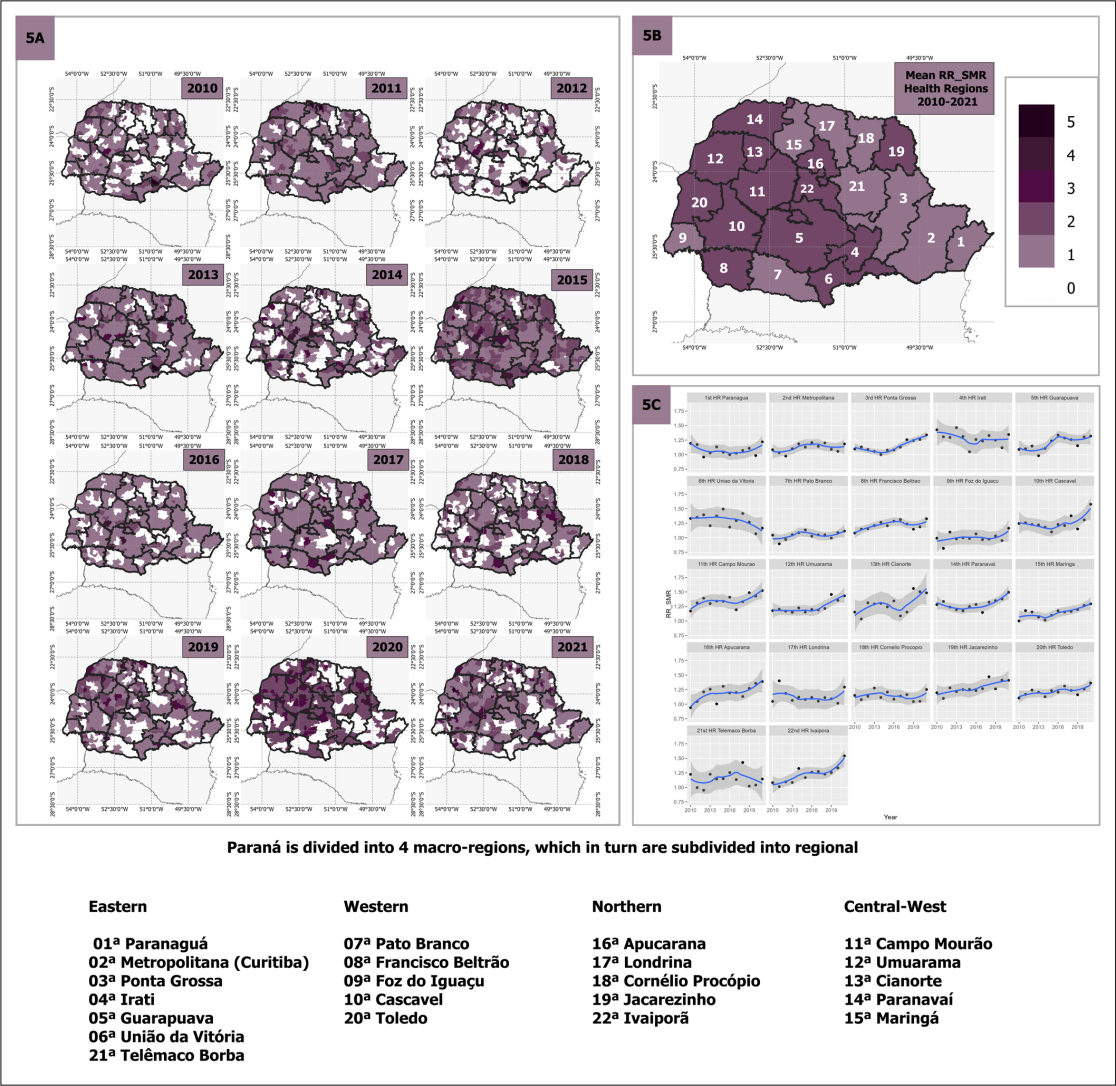
**5A** Relative risk estimates of standardized mortality ratio (SMR) per municipality and health region and scatter plot of SMR RR values per health region from 2011 to 2021 in Paraná. **5B** Average SMR RR in municipalities by health region from 2010 to 2021. **5C** Graphs with temporal presentation of average SMR RR in municipalities by health region, from 2010 to 2021. The shapefile for the basemap was obtained from the Brazilian Institute of Geography and Statistics (17).

In contrast to the findings in Figure 4A, the spatial distribution of RR for mortality in Figure 5A shows a relevant difference, indicating an elevated risk of death for most municipalities within each health region. Additionally, it is noticeable that in 2012, there was a scattered reduction in this risk, but it changed its configuration in the following year, maintaining the same pattern but intensifying the risks. In 5B we noticed that the risk of death beyond the expected moves to the west of the state. And last, in Figure 5C, regions 3, 10, 11, 12, 13, 14, 15, 16, 19, 20 and 22 presented a positive trend of deaths.

**Figure 6:**
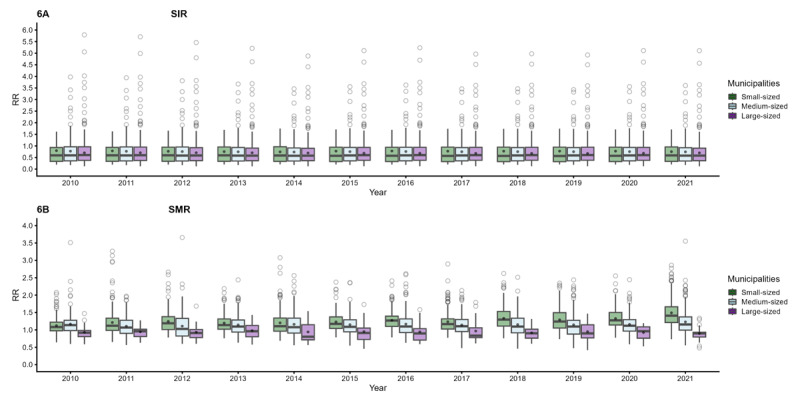
Relative risk of SIR in panel A and SMR in panel B, between 2010–2021. The mean was indicated as a black dot, in each municipality category.

Figure 6 displays 12 boxplots representing the RR for both SIR and SMR. Panel A indicates that the mean RR for hospitalization in small-sized municipalities, denoted by the circle in each boxplot, was slightly higher compared to that in medium and large municipalities. In Panel B, a similar trend is observed, with the exception of the year 2010. In essence, this figure illustrates that the mean RR for both hospitalization and mortality was consistently higher in small-sized municipalities.

## Discussion

To the best of our knowledge, this study provides an in-depth and innovative analysis of hospitalization and mortality patterns for IHD in the south region of Brazil using Bayesian modeling, thereby complementing the existing literature by corroborating and expanding on previous findings.

We identified a higher number of hospitalizations and deaths due to IHD among men, whites and individuals aged 40–59 years, except for deaths among women aged 80 years and older. The time series of SIR and SMR indicated significant variations over time in certain regions of the state and higher SIR values were observed in the eastern region while higher SMR were more diffuse but predominant in the central region of Parana state. In addition, we found an association between municipality size and IHD outcomes, suggesting that smaller municipalities face higher risks of hospitalizations and deaths due to this condition.

The study revealed that hospitalizations and mortality due to IHD are significantly related to the following variables: IPDM, as well as the size categories of small and medium municipalities. Our findings indicate that small and medium municipalities are associated with higher IHD hospitalizations and mortality rates than large municipalities, and lower IPDM was associated with higher IHD hospitalizations and mortality rates compared to those with a higher IPDM.

Our findings are in agreement with other studies that focused solely on spatial analysis in the state of Paraná, where they found that there were 32,310 deaths attributed to IHD among individuals aged 20 to 79 between 2009 and 2015 (39,40). These deaths were correlated with socioeconomic and demographic indicators such as population size and gross domestic product (39,40).

The findings above emphasize the importance of considering demographic factors, such as age, sex, and race, in understanding the disparities in IHD outcomes. Previous studies have shown that, in general, men have higher mortality rates from IHD than women. However, it is important to emphasize that incidence and mortality rates may vary according to age and geographic region (2,41,42). Silva (2018) reported that men in Paraná ranked seventh among the most affected populations by IHD in Brazil in 1990 but, by 2015, they had dropped to the 17th position (43). Furthermore, when analyzing physical inactivity as a risk factor for mortality due to IHD in the population aged above 25 years, Paraná stood out as the 10th state that experienced the most significant reduction in mortality (44). This indicates an important reduction; however, our study highlights that men continue to be the most affected population and the presence of regions that continue to face higher risks of hospitality and mortality for IHD.

De Carvalho Dutra et al. (2020) investigated the distribution of mortality from IHD associated with sociodemographic and clinical variables in the state of Paraná and identified clusters of municipalities with high mortality rates in regions 11, 12, 14 and 19, with region 11 showing particularly high rates (40). Additionally, the study revealed that the elevated mortality rates were correlated with a low index of access to cardiologists and chemical reperfusion centers. In concordance with our study, these regions presented great RR of mortality, but a decrease in hospitalization, suggesting limited access to specialized medical care as people in these regions face challenges in accessing specialized medicine.

Another ecological study conducted by Silva et al. (2022), which primarily focused on the influence of socioeconomic inequalities and environment on cardiovascular disease, found that the region 6 presented the highest RR for IHD hospitalization and mortality (44). However, our analysis revealed that sociodemographic factors were associated with higher RR for IHD when considering these factors more comprehensively. This suggests that, in addition to socioeconomic and environmental factors, a detailed examination of sociodemographic variables is essential in determining the RR for IHD in Paraná.

The present study found a reduction in the risk of IHD mortality in 2012 in the state of Paraná, which subsequently increased in the following years. Several factors might explain this trend. One of the main reasons is the implementation of the public policy called ‘Plan of Strategic Actions to Combat Non-Communicable Chronic Diseases (NCDs) in Brazil.’ This plan, initiated in 2011 and set to be developed until 2022, aims to understand the distribution, magnitude, and trends of CVDs, including IHD, their complications, and risk factors, as well as to support public health promotion policies (45). Nevertheless, the efficacy of the plan might have suffered due to the turnover of public sector managers at the onset of its implementation, coinciding with the 2012 elections and the establishment of a new local government in 2013 (45).

This study has limitations. Firstly, the reliance on historical data specifically from Paraná may restrict the generalizability of the findings to a national level, meaning that it is not nationally representative. Second, the use of secondary data can lead to delays in analysis due to challenges in obtaining up-to-date information and is susceptible to underreporting. However, it’s worth noting that the data quality has significantly improved over time, thanks to the continuous enhancements in the public information system available from the website of the Brazilian Ministry of Health. Secondly, even though patients should be treated in the same health region they reside in, it is often observed that patients seek health care in other cities, regions, or states. Unfortunately, this information cannot be traced in the public repository.

## Conclusion

In conclusion, this study revealed a higher association of IHD incidence and mortality in small and medium-sized municipalities as well as in those with a lower municipal development index, suggested that there is a deficit of care in health attention for IHD cases in small and medium-sized municipalities, which can be justified by a low distribution of health care resources, highlighting essential insights for local policy planning and decision-making by public managers. By identifying vulnerable areas and implementing targeted interventions, effective disease prevention strategies can be devised, ultimately reducing the impact of IHD on the municipalities of Paraná.

## Additional File

The additional file for this article can be found as follows:

10.5334/gh.1347.s1 Supplementary Files. The datasets and scripts utilized in this study are freely accessible.

## References

[1] Roth GA, Johnson C, Abajobir A, Abd-Allah F, Abera SF, Abyu G, et al. Global, regional, and national burden of cardiovascular diseases for 10 causes, 1990 to 2015. J Am Coll Cardiol. 2017; 70(1): 1–25. DOI: 10.1016/j.jacc.2017.04.052 28527533 PMC5491406

[2] Roth GA, Mensah GA, Johnson CO, Addolorato G, Ammirati E, Baddour LM, et al. Global burden of cardiovascular diseases and risk factors, 1990–2019: update from the GBD 2019 study. J Am Coll Cardiol. 2020; 76(25): 2982–3021. DOI: 10.1016/j.jacc.2020.11.010 33309175 PMC7755038

[3] Wang C, Sun Y, Jiang D, Wang C, Liu S. Risk-attributable burden of ischemic heart disease in 137 low- and middle-income countries from 2000 to 2019. J Am Heart Assoc. 2021; 10(19): e021024. DOI: 10.1161/JAHA.121.021024 34585592 PMC8649139

[4] Gakidou E, Afshin A, Abajobir AA, Abate KH, Abbafati C, Abbas KM, et al. Global, regional, and national comparative risk assessment of 84 behavioural, environmental and occupational, and metabolic risks or clusters of risks, 1990–2016: a systematic analysis for the Global Burden of Disease Study 2016. Lancet. 2017; 390(10100): 1345–1422. DOI: 10.1016/S0140-6736(17)32366-8 28919119 PMC5614451

[5] Wang F, Yu Y, Mubarik S, Zhang Y, Liu X, Cheng Y, et al. Global burden of ischemic heart disease and attributable risk factors, 1990–2017: a secondary analysis based on the global burden of disease study 2017. Clin Epidemiol. 2021; 13: 859–870. DOI: 10.2147/CLEP.S317787 34584461 PMC8464307

[6] Collaborators GB, Ärnlöv J. Global burden of 87 risk factors in 204 countries and territories, 1990–2019: a systematic analysis for the Global Burden of Disease Study 2019. Lancet. 2020; 396(10258): 1223–1249. DOI: 10.1016/S0140-6736(17)32366-8 33069327 PMC7566194

[7] Finegold JA, Asaria P, Francis DP. Mortality from ischaemic heart disease by country, region, and age: statistics from World Health Organization and United Nations. Int J Cardiol. 2013; 168(2): 934–945. DOI: 10.1016/j.ijcard.2012.10.046 23218570 PMC3819990

[8] Oliveira GMM, Brant LCC, Polanczyk CA, Malta DC, Biolo A, Nascimento BR, et al. Estatística Cardiovascular–Brasil 2021. Arq Bras Cardiol. 2022; 118(1): 115–373. DOI: 10.36660/abc.20211012 35195219 PMC8959063

[9] Oliveira GMM, Brant LCC, Polanczyk CA, Biolo A, Nascimento BR, Malta DC, et al. Cardiovascular statistics–Brazil 2020. Arq Bras Cardiol. 2020; 115: 308–439. DOI: 10.36660/abc.20200812 33027364 PMC9363085

[10] Brant LCC, Nascimento BR, Veloso GA, Gomes CS, Polanczyk C, Oliveira GMM, et al. Burden of Cardiovascular diseases attributable to risk factors in Brazil: data from the Global Burden of Disease 2019 study. Rev Soc Bras Med Trop. 2022; 55: e0263–2021. DOI: 10.1590/0037-8682-0263-2021 PMC9009428 35107526

[11] Wang Y, Du Q, Ren F, Liang S, Lin DN, Tian Q, et al. Spatio-temporal variation and prediction of ischemic heart disease hospitalizations in Shenzhen, China. Int J Environ Res Public Health. 2014; 11(5): 4799–4824. DOI: 10.3390/ijerph110504799 24806191 PMC4053872

[12] Kim D, Sarker M, Vyas P. Role of spatial tools in public health policymaking of Bangladesh: opportunities and challenges. J Health Popul Nutr. 2016; 35: 8. DOI: 10.1186/s41043-016-0045-1 26922788 PMC5026007

[13] Forouzanfar MH, Moran AE, Flaxman AD, Roth G, Mensah GA, Ezzati M, et al. Assessing the global burden of ischemic heart disease, part 2: analytic methods and estimates of the global epidemiology of ischemic heart disease in 2010. Glob Heart. 2012; 7(4): 331–342. DOI: 10.1016/j.gheart.2012.10.003 23505617 PMC3595103

[14] Martinez-Beneito MA, Vergara-Hernández C, Botella-Rocamora P, Corpas-Burgos F, Pérez-Panadés J, Zurriaga Ó, et al. Geographical variability in mortality in urban areas: a joint analysis of 16 causes of death. Int J Environ Res Public Health. 2021; 18(11): 564. DOI: 10.3390/ijerph18115664 34070635 PMC8197960

[15] Okui T, Matoba T, Nakashima N. The association between the socioeconomic deprivation level and ischemic heart disease mortality in Japan: an analysis using municipality-specific data. Epidemiol Health. 2022; 44: e2022059. DOI: 10.4178/epih.e2022059 35879856 PMC9754915

[16] Cuschieri S. The STROBE guidelines. Saudi J Anaesth. 2019; 13(Suppl 1): S31–34. DOI: 10.4103/sja.SJA_543_18 30930717 PMC6398292

[17] Instituto Brasileiro de Geografia e Estatística (IBGE). Cidades; 2023 [cited 2023 Aug 6]. Available from: https://cidades.ibge.gov.br.

[18] Santos L. Região de saúde e suas redes de atenção: modelo organizativo-sistêmico do SUS. Ciênc Saúde Colet. 2017; 22: 1281–1289. DOI: 10.1590/1413-81232017224.26392016 28444052

[19] Guimarães RB. Regiões de saúde e escalas geográficas. Cad Saude Publica. 2005; 21: 1017–25. DOI: 10.1590/S0102-311X2005000400004 16021239

[20] Paim J, Travassos C, Almeida C, Bahia L, Macinko J. The Brazilian health system: history, advances, and challenges. Lancet. 2011; 377(9779): 1778–1797. DOI: 10.1016/S0140-6736(11)60054-8 21561655

[21] Silva LL, de Carvalho Dutra A, de Andrade L, Iora PH, Rodrigues Ramajo GL, Peres Gualda IA, et al. Emergency care gap in Brazil: geographical accessibility as a proxy of response capacity to tackle COVID-19. Front Public Health. 2021; 9: 740284. DOI: 10.3389/fpubh.2021.740284 34869155 PMC8634954

[22] Pereira CCA, Soares FRG, Machado CJ, Frio GS, Alves LC, Herkrath FJ, et al. Development of an index to assess COVID-19 hospital care installed capacity in the 450 Brazilian health regions. Disaster Med Public Health Prep. 2022: 1–8. DOI: 10.1017/dmp.2022.214 PMC9588413 35993500

[23] Arnett DK, Khera A, Blumenthal RS. 2019 ACC/AHA guideline on the primary prevention of cardiovascular disease: part 1, lifestyle and behavioral factors. JAMA Cardiol. 2019; 4(10): 1043–1044. DOI: 10.1161/CIR.0000000000000678 31365022

[24] Kaptoge S, Pennells L, De Bacquer D, Cooney MT, Kavousi M, Stevens G, et al. World Health Organization cardiovascular disease risk charts: revised models to estimate risk in 21 global regions. Lancet Glob Health. 2019; 7(10): e1332–e1345. DOI: 10.1016/S2214-109X(19)30318-3 31488387 PMC7025029

[25] Departamento de Informática do Sistema Único de Saúde (DATASUS). 2023 [cited 2023 Aug 6]. Available from: https://datasus.saude.gov.br.

[26] Instituto Brasileiro de Geografia e Estatística (IBGE). Sistema IBGE de recuperação automática-SIDRA. Várias tabelas; 2008 [cited 2023 Aug 6].

[27] Instituto Brasileiro de Geografia e Estatística (IBGE). Pesquisa nacional por amostra de domicílios contínua. Estatísticas sociais; 2022 [cited 2023 Aug 6]. Available from: https://www.ibge.gov.br/.

[28] Dos Santos M, Oliveira Penteado J, de Lima Brum R, da Silva Bonifácio A, Florêncio Ramires P, de Franceschi Gariboti D, et al. Ethnic/racial disparity in mortality from COVID-19: data for the Year 2020 in Brazil. Spat Demogr. 2023; 11(1): 1–17. Epub 2023 Jan 16. PubMed PMID: 36685786. PubMed Central PMCID: PMC9841953. DOI: 10.1007/s40980-022-00112-2 PMC9841953 36685786

[29] Instituto Paranaense de Desenvolvimento Econômico e Social (IPARDES). Estatísticas econômicas do estado do Paraná; 2021 [cited 2023 Aug 6]. Available from: https://www.ipardes.gov.br/estatisticas/economicas.

[30] Moraga P, Dean C, Inoue J, Morawiecki P, Noureen SR, Wang F. Bayesian spatial modelling of geostatistical data using INLA and SPDE methods: a case study predicting malaria risk in Mozambique. Spat Spatiotemporal Epidemiol. 2021; 39: 100440. Epub 2021 Aug 3. PubMed PMID: 34774255. DOI: 10.1016/j.sste.2021.100440 34774255

[31] Arango-Londoño D, Ortega-Lenis D, Moraga P, Torres M, Rodríguez-Cortés FJ. Spatial modeling and socioeconomic inequities of COVID-19 in the urban area of the city of Cali, Colombia. Spat Spatiotemporal Epidemiol. 2023; 44: 100561. Epub 2022 Dec 16. PubMed PMID: 36707197. PubMed Central PMCID: PMC9756648. DOI: 10.1016/j.sste.2022.100561 36707197 PMC9756648

[32] Bernardinelli L, Clayton D, Pascutto C, Montomoli C, Ghislandi M, Songini M. Bayesian analysis of space-time variation in disease risk. Stat Med. 1995; 14(2122): 2433–43. PubMed PMID: 8711279. DOI: 10.1002/sim.4780142112 8711279

[33] Becher H, Winkler V. Estimating the standardized incidence ratio (SIR) with incomplete follow-up data. BMC Med Res Methodol. 2017; 17(1): 55. Epub 2017 Apr 12. PubMed PMID: 28403811. PubMed Central PMCID: PMC5389158. DOI: 10.1186/s12874-017-0335-3 28403811 PMC5389158

[34] Rue H, Martino S, Chopin N. Approximate Bayesian inference for latent Gaussian models by using integrated nested Laplace approximations. J R Stat Soc Series B Stat Methodol. 2009; 71(2): 319–92. DOI: 10.1111/j.1467-9868.2008.00700.x

[35] Sumetsky N, Mair C, Anderson S, Gruenewald PJ. A spatial partial differential equation approach to addressing unit misalignments in Bayesian poisson space-time models. Spat Spatiotemporal Epidemiol. 2020; 33: 100337. Epub 2020 Mar 6. PubMed PMID: 32370937. PubMed Central PMCID: PMC7499432. DOI: 10.1016/j.sste.2020.100337 32370937 PMC7499432

[36] Yoo W, Mayberry R, Bae S, Singh K, He QP, Lillard JW Jr. A study of effects of multicollinearity in the multivariable analysis. Int J Appl Sci Technol. 2014; 4(5): 9–19. PubMed PMID: 25664257. PubMed Central PMCID: PMC4318006. DOI: 10.1007/978-0-387-35973-1_1064 25664257 PMC4318006

[37] Chan JY, Leow SM, Bea KT, Cheng WK, Phoong SW, Hong ZW, et al. Mitigating the multicollinearity problem and its machine learning approach: a review. Mathematics. 2022; 10(8). DOI: 10.3390/math10081224

[38] Hugentobler M. Quantum GIS. In: Shekhar S, Xiong H, editors. Encyclopedia of GIS. Boston, MA: Springer US; 2008. pp. 935–939. DOI: 10.1007/978-0-387-35973-1_1064

[39] Bergamini M, Iora PH, Rocha TA, Tchuisseu YP, Dutra ADC, Scheidt JF, et al. Mapping risk of ischemic heart disease using machine learning in a Brazilian state. PLoS One. 2020; 15(12): e0243558. DOI: 10.1371/journal.pone.0243558 33301451 PMC7728276

[40] De Carvalho Dutra A, Silva LL, Pedroso RB, Tchuisseu YP, da Silva MT, Bergamini M, et al. The impact of socioeconomic factors, coverage and access to health on heart ischemic disease mortality in a Brazilian Southern state: a geospatial analysis. Glob Heart. 2021; 16(1): 25. DOI: 10.5334/gh.770 33598385 PMC7824986

[41] Puzzi M, Massago M, Gabella JL, de Oliveira SB, Dos Santos DAM, Carignano FSN, et al. Mortality in women with coronary artery disease in Paraná State, Brazil: a Bayesian spatiotemporal analysis. Glob Heart. 2024; 19(1): 8. DOI: 10.5334/gh.1297 38312999 PMC10836177

[42] Baena CP, Chowdhury R, Schio NA, Sabbag AE Jr, Guarita-Souza LC, Olandoski M, et al. Ischaemic heart disease deaths in Brazil: current trends, regional disparities and future projections. Heart. 2013; 99(18): 1359–1364. Epub 2013 Jul 25. PubMed PMID: 23886609. DOI: 10.1136/heartjnl-2013-303617 23886609

[43] Silva DAS, Malta DC, Souza MFM, Naghavi M. Burden of ischemic heart disease mortality attributable to physical inactivity in Brazil. Rev Saude Publica. 2018; 52: 72. Epub 2018 Jul 26. PubMed PMID: 30066811. PubMed Central PMCID: PMC6063711. DOI: 10.11606/S1518-8787.2018052000413 30066811 PMC6063711

[44] Silva ID, Wikuats CFH, Hashimoto EM, Martins LD. Effects of environmental and socioeconomic inequalities on health outcomes: a multi-region time-series study. Int J Environ Res Public Health. 2022; 19(24): 16521. Epub 2022 Dec 9. PubMed PMID: 36554402. PubMed Central PMCID: PMC9778807. DOI: 10.3390/ijerph192416521 36554402 PMC9778807

[45] Ministério da Saúde. Plano de ações estratégicas para o enfrentamento das doenças crônicas não transmissíveis (DCNT) no Brasil 2011–2022 [cited 2023 Aug 6]. Brasília: Ministério da Saúde; 2011.

