# Supplementary material for: Bayesian Modeling and Estimation of Spatial Risk for Hospitalization and Mortality from Ischemic Heart Disease in Paraná, Brazil
Source: Glob Heart. 2024 Aug 5;19(1):63. doi: 10.5334/gh.1347 (PMC11312845; doi:10.5334/gh.1347)
Supplement: Supplementary Files. — The datasets and scripts utilized in this study are freely accessible. [file gh-19-1-1347-s1.pdf]

Bayesian modeling and estimation of spatial risk for hospitalization and mortality from ischemic heart disease in Paraná state, Brazil

Corresponding author:
Professor PhD Luciano de Andrade
Department of Medicine, State University of Maringa
Post-Graduation Program in Health Sciences
landrade@uem.br

Data availability

All data are publicly and freely available from the Brazilian Health System Informatics Department (DATASUS), and cartographic base data for all municipalities in Paraná State were obtained from the Paraná branch of the Brazilian Institute of Geography and Statistics (IBGE). Table below lists all datasets accessed and utilized in this study. The database and script utilized in this study can be found in the online repository: https://doi.org/10.6084/m9.figshare.25007108.v1.

**Source**	**Variables**	**Link**
DATASUS - Mortality Information System (SIM)	Mortality<br>• sex (Men,Women),<br>• race (White,Non-white)<br>• Age groups (40-59,60-69,70-79, 80+)	https://datasus.saude.gov.br/
DATASUS - Hospitalizations Information System (SIH)	Hospitalizations<br>• sex (Men,Women),<br>• race (White,Non-white)<br>• Age groups (40-59,60-69,70-79, 80+)	https://datasus.saude.gov.br/
IBGE - Brazilian Institute of Geography and Statistics	Population by<br>• sex (Men,Women),<br>• race (White,Non-white)<br>• Age groups (40-59,60-69,70-79, 80+)	https://www.ibge.gov.br/
IBGE - Brazilian Institute of Geography and Statistics	Cartographic base data for all municipalities and regional health in Paraná State	https://www.ibge.gov.br/

Here, we provide the script for reproducibility.

```
install.packages("INLA", repos = "https://inla.r-inla-download.org/R/stable", dep = TRUE)
library("sf")
library("tidyverse")
```

```
library(SpatialEpiApp)
library(SpatialEpi)
```

Load the database

#head (data)

county	name	year	age	race	gender	population	mortality	hospitalizations
410010	ABATIÁ	2010	40.59	w	m	734	0	3
410010	ABATIÁ	2010	40.59	o	m	315	1	0
410010	ABATIÁ	2010	40.59	w	f	708	0	1
410010	ABATIÁ	2010	40.59	o	f	304	0	0
410010	ABATIÁ	2010	60.69	w	m	209	2	2
410010	ABATIÁ	2010	60.69	o	m	89	2	0

#head (variables)

county	IPDM	Small-size municipalities	Medium-size municipalities	Large-size municipalities
410010	0.6003	1	0	0
410020	0.6127	1	0	0
410030	0.5264	1	0	0
410040	0.5565	0	0	1
410045	0.6446	1	0	0
410050	0.6762	0	1	0

#map (PR)

```
library(sp)
rownames(d) <- d$id
map <- merge(map, d, by.x = "county", by.y = "id")
head(map@data)
colnames(d)
str(map@data)
```

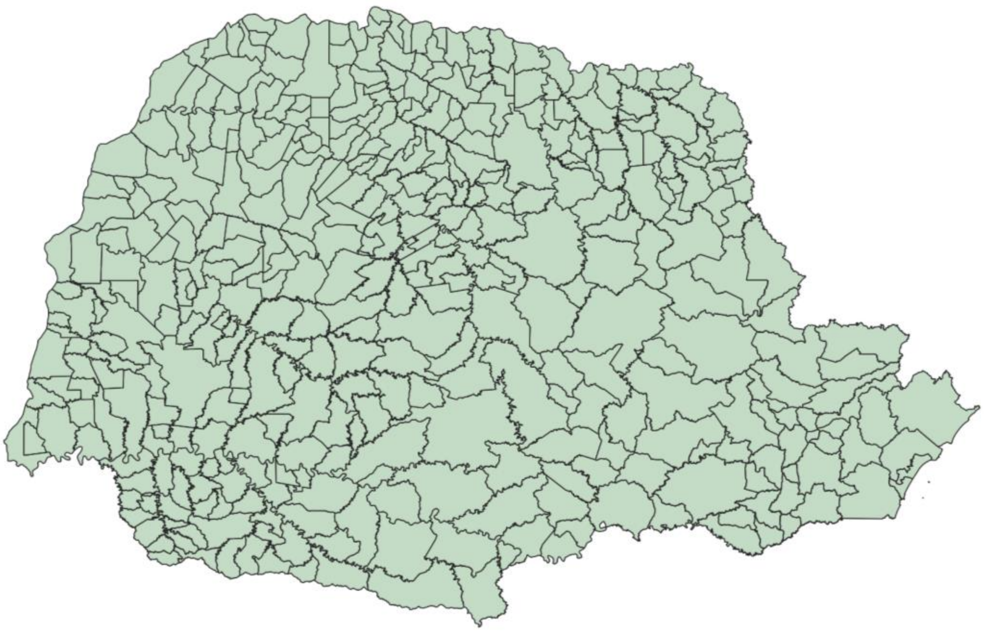

Data Organization

We calculated the observed and expected counts, as well as the SMRs and SIRs for each municipality and year, and created a data frame with the following variables:

County:ID of each municipality,

Year: year,

Y: Observed number of cases for the municipality and year,

E: Expected number of cases for the municipality and year,

SMR: observed mortality number / expected mortality number,

SIR: observed hospitalizations number / expected hospitalizations number.

Observed Cases

We obtain the number of cases for all strata combined in each municipality and year by aggregating the data by municipality. To do this, we use the aggregate() function specifying the cases vector, the list of grouping elements such as list(county = data$name, year = data$year), and the function to be applied to subsets of data, which is the mean. We also set the names of the returned data frame as county, year, and Y.

Code and script

```
#Observed cases mortality
d <- aggregate(x = banco$mortality, by = list(county = banco$county), FUN = mean)
names(d) <- c("id", "Y")

#Observed cases hospitalizations
d <- aggregate(x = banco$hospitalizations, by = list(county = banco$county), FUN = mean)
names(d) <- c("id", "Y")
```

**id**	**Y**
**410010**	**1.2187**
**410020**	**1.7395**
**410030**	**2.6666**
**410040**	**19.4635**
**410045**	**0.5937**
**410050**	**2.4479**

```
# Expected cases mortality
banco <- banco[order(banco$county, banco$race,
               banco$gender, banco$age), ]
population <-banco$population
cases <- banco$mortality
n.strata <- 16
E <- expected(population, cases, n.strata)
d$E <- E[match(d$id, unique(banco$county))]

table(is.na(d$E))


# Expected cases hospitalizations
banco <- banco[order(banco$county, banco$race,
               banco$gender, banco$age), ]
population <-banco$population
cases <- banco$hospitalizations
n.strata <- 16
E <- expected(population, cases, n.strata)
d$E <- E[match(d$id, unique(banco$county))]

table(is.na(d$E))
```

**id**	**Y**	**E**
**410010**	**1.2187**	**26.2206**
**410020**	**1.7395**	**10.1291**
**410030**	**2.6666**	**5.1360**
**410040**	**19.4635**	**26.1302**

**410045**	**0.5937**	**8.6864**
**410050**	**2.4479**	**3.8677**

Statistical analysis using INLA

This model assumes that the number of observed cases in a specific municipality in a given year follows a Poisson distribution.

$$Oij{\sim}Poisson\ (nij\Theta ij)$$

The formula "Oij ~ Poisson (nijΘij)" describes a statistical relationship being used to model the number of observed cases (Oij) in a specific context, such as epidemiology or health data analysis. Let's understand the meaning of the components of this formula:

Oij: Represents the number of observed cases in a specific context, like a municipality, year, or another unit of analysis. It is the variable we are interested in modeling or understanding.

Poisson: Refers to the Poisson distribution, which is a discrete probability distribution used to model the number of rare or discrete events occurring in a fixed time or space interval. In this context, we assume that the number of observed cases follows a Poisson distribution.

nij: Represents the number of people or the population at risk in the same context where we are counting the cases. In other words, it is the number of individuals who are potentially subject to the event we are studying.

Θij: Is the parameter of the Poisson distribution and represents the rate of occurrence of the event in question in the specific context (municipality, year.). It is the value we are trying to estimate or model.

Therefore, the formula "Oij ~ Poisson (nijΘij)" indicates that we are modeling the number of observed cases (Oij) as a random variable following a Poisson distribution, where the rate of occurrence (Θij) is multiplied by the population at risk (nij) to determine the probability of observing a specific number of cases.

The number of observed cases is influenced by the expected number of cases and the relative risk of that specific municipality and year. This model allows each area to have its own intercept and linear trend, considering the specific characteristics of each municipality.

$$log(\Theta ij) = (\mu + \phi i) + (\beta + \delta i)tj.$$

log(Θij): Refers to the natural logarithm of the occurrence rate (Θij) of the event under study. The logarithmic transformation is common in statistical models to stabilize variability and ensure that values are positive.

μ: Represents an overall intercept or baseline of the occurrence rate. It is a constant parameter that captures the average level of the occurrence rate across the entire population or context.

ϕi: Represents a specific random effect for each municipality (or other unit of analysis) denoted by i. These random effects capture spatial variation or unexplained variation beyond the overall intercept. They reflect the unique characteristics of each unit of analysis.

β: Represents the overall effect of the time variable (tj) on the occurrence rate. It is a parameter that models the average temporal trend across all contexts.

δi: Represents the specific effect of each municipality for the time variable (tj). These effects capture the specific temporal variation of each unit of analysis.

tj: Is the time variable that may represent different time points at which data were collected. It is used to model temporal variations in the occurrence rate.

```
# SMR
d$SMR <- d$Y/d$E

# SIR
d$SIR <- d$Y/d$E

map <- merge(map, d, by.x = "county", by.y = "id")
head(map@data)
colnames(d)
str(map@data)
```

#SMR

```
# Mapping variables
library(leaflet)
l <- leaflet(map) %>% addTiles()
pal <- colorNumeric(palette = "YlOrRd", domain = map$SMR)
l %>% addPolygons(color = "grey", weight = 1, fillColor = ~pal(SMR), fillOpacity = 0.5)
%>%
  addLegend(pal = pal, values = ~SMR, opacity = 0.5, title = "SMR", position =
"bottomright")


labels <- sprintf("<strong> %s </strong> <br/> Observed: %s <br/> Expected: %s <br/>
INDEX1021: %s <br/> SMR:%s <br/> Small_sized:%s <br/>
                 Medium_sized:%s <br/> Lager_sized:%s",
                 map$id, map$Y, map$E, map$INDEX1021, map$SMR,map$Small_sized,
                 map$Medium_sized, map$Lager_sized) %>%
  lapply(htmltools::HTML)
l %>% addPolygons(color = "grey", weight = 1, fillColor = ~pal(SMR), fillOpacity = 0.5,
                 highlightOptions = highlightOptions(weight = 4), label = labels,
                 labelOptions = labelOptions(style = list("font-weight" = "normal",
                                                          padding = "3px 8px"),
                                             textsize = "15px", direction = "auto")) %>%
  addLegend(pal = pal, values = ~SMR, opacity = 0.5, title = "SMR", position =
"bottomright")
```

```
# Neighbourhood matrix
library(spdep)
library(foreach)
library(INLA)
nb <- poly2nb(map)
head(nb)
nb2INLA("map.adj", nb)
g <- inla.read.graph(filename = "map.adj")


# Inference using INLA
map$re_u <- 1:nrow(map@data)
map$re_v <- 1:nrow(map@data)
map@data$Y <- as.integer(map@data$Y)
colnames(d)

formula <- Y ~ offset(log(POP))+INDEX1021+Small_sized+Medium_sized+Lager_sized
model1 <- glm(formula=formula, family = "poisson", data = map@data)
res <- inla(formula, family = "poisson", data = map@data, E = model1$fitted.values,
            control.predictor = list(compute = TRUE))
FixedEffects(res)
summary(res)
summary(model1)
summary(model1)$coefficients
model1$expected_values
model1
print(model1)
names(model1)
str(map@data)

table(is.na(map@data$Lager_sized))

# Results
summary(res)
library(ggplot2)
modelo <- lm(Y ~ INDEX1021+Small_sized+Medium_sized+Lager_sized, data =
map@data)
vif_result <- vif(model1)
print(vif_result)

formula <- Y ~ offset(log(E))+INDEX1021+Small_sized+Medium_sized+Lager_sized
model1 <- glm(formula=formula, family = "poisson", data = map@data)
summary(model1)
summary(model1)$coefficients
model1$expected_values
model1
library(glmmTMB)
m <- glm(Y ~ INDEX1021+Small_sized+Medium_sized+Lager_sized, family = poisson,
data = map@data)

performance::check_overdispersion(m)


m <- glmmTMB(
  Y ~ INDEX1021+Small_sized+Medium_sized+Lager_sized + (1 |CODIBGE),
  family = poisson,
```

```
  data = map@data
)
performance::check_overdispersion(m)

head(res$summary.fitted.values)
names(res)
map$RR <- res$summary.fitted.values[, "mean"]
map$LL <- res$summary.fitted.values[, "0.025quant"]
map$UL <- res$summary.fitted.values[, "0.975quant"]


# Mapping disease risk
pal <- colorNumeric(palette = "YlOrRd", domain = map$RR)
labels <- sprintf("<strong> %s </strong> <br/> Observed: %s <br/> Expected: %s <br/>
INDEX1021: %s <br/> SMR:%s <br/> Small_sized:%s <br/>
                Medium_sized:%s <br/> Lager_sized:%s <br/> RR: %s (%s, %s)",
                map$id, map$Y, map$E, map$INDEX1021, map$SMR,map$Small_sized,
                map$Medium_sized, map$Lager_sized,
                round(map$SMR, 2),round(map$RR, 2), round(map$LL, 2), round(map$UL, 2))
%>%
  lapply(htmltools::HTML)
labels <- unlist(labels)
leaflet(map) %>% addTiles() %>%
  addPolygons(color = "grey", weight = 1, fillColor = ~pal(RR), fillOpacity = 0.5,
             highlightOptions = highlightOptions(weight = 4), label = labels,
             labelOptions = labelOptions(style = list("font-weight" = "normal",
                                                      padding = "3px 8px"),
                                         textsize = "15px", direction = "auto")) %>%
  addLegend(pal = pal, values = ~RR, opacity = 0.5, title = "RR", position = "bottomright")

# Map disease risk with the same scale as map of SMR
pal <- colorNumeric(palette = "YlOrRd", domain = map$SMR)
leaflet(map) %>% addTiles() %>%
  addPolygons(color = "grey", weight = 1, fillColor = ~pal(RR), fillOpacity = 0.5,
             highlightOptions = highlightOptions(weight = 4), label = labels,
             labelOptions = labelOptions(style = list("font-weight" = "normal",
                                                      padding = "3px 8px"),
                                         textsize = "15px", direction = "auto")) %>%
  addLegend(pal = pal, values = ~RR, opacity = 0.5, title = "RR", position = "bottomright")

# Range of values of SMRs and RRs

range(map@data$SMR)
range(map@data$RR)

head(map@data)

summary(d$Y)
summary(d$E)


pacman::p_load(pacman, microdatasus, dplyr, rio, datasets, plyr)
export(map@data,"mapSMR1021_teste.csv")
```

#SIR

```
# Mapping variables
library(leaflet)
l <- leaflet(map) %>% addTiles()
pal <- colorNumeric(palette = "YlOrRd", domain = map$SIR)
l %>% addPolygons(color = "grey", weight = 1, fillColor = ~pal(SIR), fillOpacity = 0.5) %>%
  addLegend(pal = pal, values = ~SIR, opacity = 0.5, title = "SIR", position = "bottomright")

labels <- sprintf("<strong> %s </strong> <br/> Observed: %s <br/> Expected: %s <br/>
INDEX1021: %s <br/> SIR:%s <br/> Small_sized:%s <br/>
                 Medium_sized:%s <br/> Lager_sized:%s",
                 map$id, map$Y, map$E, map$INDEX1021, map$SIR,map$Small_sized,
                 map$Medium_sized, map$Lager_sized) %>%
  lapply(htmltools::HTML)
l %>% addPolygons(color = "grey", weight = 1, fillColor = ~pal(SIR), fillOpacity = 0.5,
                 highlightOptions = highlightOptions(weight = 4), label = labels,
                 labelOptions = labelOptions(style = list("font-weight" = "normal",
                                                     padding = "3px 8px"),
                                          textsize = "15px", direction = "auto")) %>%
  addLegend(pal = pal, values = ~SIR, opacity = 0.5, title = "SIR", position = "bottomright")

# Neighbourhood matrix
library(spdep)
library(foreach)
library(INLA)
nb <- poly2nb(map)
head(nb)
nb2INLA("map.adj", nb)
g <- inla.read.graph(filename = "map.adj")

# Inference using INLA
map$re_u <- 1:nrow(map@data)
map$re_v <- 1:nrow(map@data)
map@data$Y <- as.integer(map@data$Y)
colnames(d)
formula <- Y ~ INDEX1021+Small_sized+Medium_sized+Lager_sized+f(re_u, model =
"besag", graph = g) + f(re_v, model = "iid")


res <- inla(formula, family = "poisson", data = map@data, E = model1$fitted.values,
           control.predictor = list(compute = TRUE))

# Results
summary(res)
library(ggplot2)

modelo <- lm(Y ~ INDEX1021+Small_sized+Medium_sized+Lager_sized, data =
map@data)
vif_result <- vif(model1)
print(vif_result)

formula <- Y ~ offset(log(POP))+INDEX1021+Small_sized+Medium_sized+Lager_sized
model1 <- glm(formula=formula, family = "poisson", data = map@data)
res <- inla(formula, family = "poisson", data = map@data, E = model1$fitted.values,
           control.predictor = list(compute = TRUE))
summary(res)
summary(model1)
summary(model1)$coefficients
```

```
model1$expected_values
model1
print(model1)
names(model1)

library(glmmTMB)
m <- glm(Y ~ INDEX1021+Small_sized+Medium_sized+Lager_sized, family = poisson,
data = map@data)

performance::check_overdispersion(m)


m <- glmmTMB(
  Y ~ INDEX1021+Small_sized+Medium_sized+Lager_sized + (1 |CODIBGE),
  family = poisson,
  data = map@data
)
performance::check_overdispersion(m)


head(res$summary.fitted.values)
names(res)
map$RR <- res$summary.fitted.values[, "mean"]
map$LL <- res$summary.fitted.values[, "0.025quant"]
map$UL <- res$summary.fitted.values[, "0.975quant"]

# Instale e carregue os pacotes necessários
install.packages("INLA")
library(INLA)

# Ajuste o modelo de regressão de Poisson usando o INLA
# Ajuste do modelo usando o INLA
inla.model <- inla(formula = formula, data = map@data, family = "poisson")

# Extraia os coeficientes estimados do modelo
coefficients <- inla.model$summary.fixed[, "mean"]  # Coeficientes estimados

# Calcule os riscos relativos
RR <- exp(coefficients)  # Riscos relativos

# Exiba os riscos relativos estimados
print(RR)


# Mapping disease risk
pal <- colorNumeric(palette = "YlOrRd", domain = map$RR)
labels <- sprintf("<strong> %s </strong> <br/> Observed: %s <br/> Expected: %s <br/>
INDEX1021: %s <br/> SIR:%s <br/> Small_sized:%s <br/>
                  Medium_sized:%s <br/> Lager_sized:%s <br/> RR: %s (%s, %s)",
                  map$id, map$Y, map$E, map$INDEX1021, map$SIR,map$Small_sized,
                  map$Medium_sized, map$Lager_sized,
                  round(map$SIR, 2),round(map$RR, 2), round(map$LL, 2), round(map$UL, 2))
%>%
  lapply(htmltools::HTML)
labels <- unlist(labels)
leaflet(map) %>% addTiles() %>%
  addPolygons(color = "grey", weight = 1, fillColor = ~pal(RR), fillOpacity = 0.5,
```

```
            highlightOptions = highlightOptions(weight = 4), label = labels,
            labelOptions = labelOptions(style = list("font-weight" = "normal",
                                                      padding = "3px 8px"),
                                        textsize = "15px", direction = "auto")) %>%
  addLegend(pal = pal, values = ~RR, opacity = 0.5, title = "RR", position = "bottomright")

# Map disease risk with the same scale as map of SIR
pal <- colorNumeric(palette = "YlOrRd", domain = map$SIR)
leaflet(map) %>% addTiles() %>%
  addPolygons(color = "grey", weight = 1, fillColor = ~pal(RR), fillOpacity = 0.5,
            highlightOptions = highlightOptions(weight = 4), label = labels,
            labelOptions = labelOptions(style = list("font-weight" = "normal",
                                                      padding = "3px 8px"),
                                        textsize = "15px", direction = "auto")) %>%
  addLegend(pal = pal, values = ~RR, opacity = 0.5, title = "RR", position = "bottomright")

# Range of values of SIRs and RRs

range(map@data$SIR)
range(map@data$RR)

head(map@data)

summary(d$Y)
summary(d$E)


pacman::p_load(pacman, microdatasus, dplyr, rio, datasets, plyr)
export(map@data,"mapSIR1021_teste.csv")
```

From here, the results were exported along with their respective shapefiles and plotted in QGIS.
